# Cricothyroid Approximation in Trans Women With Type A Cricothyroid Joints

**DOI:** 10.1002/lary.70105

**Published:** 2025-09-01

**Authors:** Claudio Storck, Anna Schaufelbuehl, Carla Mueller, Flurin Honegger

**Affiliations:** ^1^ Department of Otorhinolaryngology, Head and Neck Surgery, Department of Phoniatrics University Hospital Basel Basel Switzerland

**Keywords:** cricothyroid approximation, cricothyroid joint, pitch‐raising surgery, trans women, voice feminization

## Abstract

**Objective:**

In trans women, low‐pitched voice can be raised by cricothyroid approximation (CTA). The aim of the study was to analyze voice outcomes in trans women with Type A cricothyroid joints (CTJs) over a period of 5 years.

**Study Design:**

Prospective cohort study.

**Methods:**

Thirty‐five trans women were included in the study after high‐resolution computed tomography evaluation revealed a Type A CTJ (Type A: well‐defined facet; B: no definable facet; C: flat cartilage surface). All had voice therapy before CTA. Additionally, voice assessment (mean speaking level [MSL], loudness, vocal range of speaking voice, Trans Women Voice Questionnaire [TWVQ]) was performed before and after voice therapy, and after CTA at 4 weeks, 6 months, and 1 year, and then annually for 5 years.

**Results:**

MSL rose with voice therapy from 134 to 150 Hz. With CTA, the MSL increased to 184 Hz 4 weeks postoperatively and to 199 Hz 6 months postoperatively. The MSL has remained stable at 203–209 Hz for 5 years. The TWVQ score decreased from 91 to 84 patients with voice therapy. After CTA, it decreased to 50 patients and has remained stable for 5 years.

**Conclusion:**

CTA in trans women with Type A CTJ is a valuable technique for elevating MSL, providing stable results for 5 years. Therefore, CTA should only be performed in patients with Type A CTJs who desire higher pitched voices.

**Level of Evidence:**

Level 4.

## Graphical abstract

Cricothyroid approximation is a proven technique to increase the speaking voice in trans women. We were able to show in a group of patients that this operation increases the speaking voice in trans women with a Type A cricothyroid joint (CTJ) and remains at a stable high level for several years. Thus, if a cricothyroid approximation is indicated, an HRCT should be performed first to confirm a Type A CTJ.

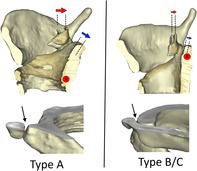

## 1 Introduction

The voice is an important factor in our identity. However, this is very problematic for patients with gender dysphoria. Transgender women, or trans women, who were assigned male at birth but identify as female, report severe limitations due to this dysphoria [1]. In view of the fact that the voice transmits information about gender, it is understandable that transfeminine vocal gender dysphoria also arises [2].

Feminizing the voice is one of the biggest hurdles for trans women during their transition [3]. It is therefore important to address voice adjustment in trans women at an early stage of an interdisciplinary approach to treatment. Various options are available for this. First and foremost, voice therapy with trained speech‐language pathologists can be used to feminize the voice. In addition to raising the frequency of the voice, this also involves melody, prosody, emphasis, and changing the vocabulary [3].

**FIGURE 1:**
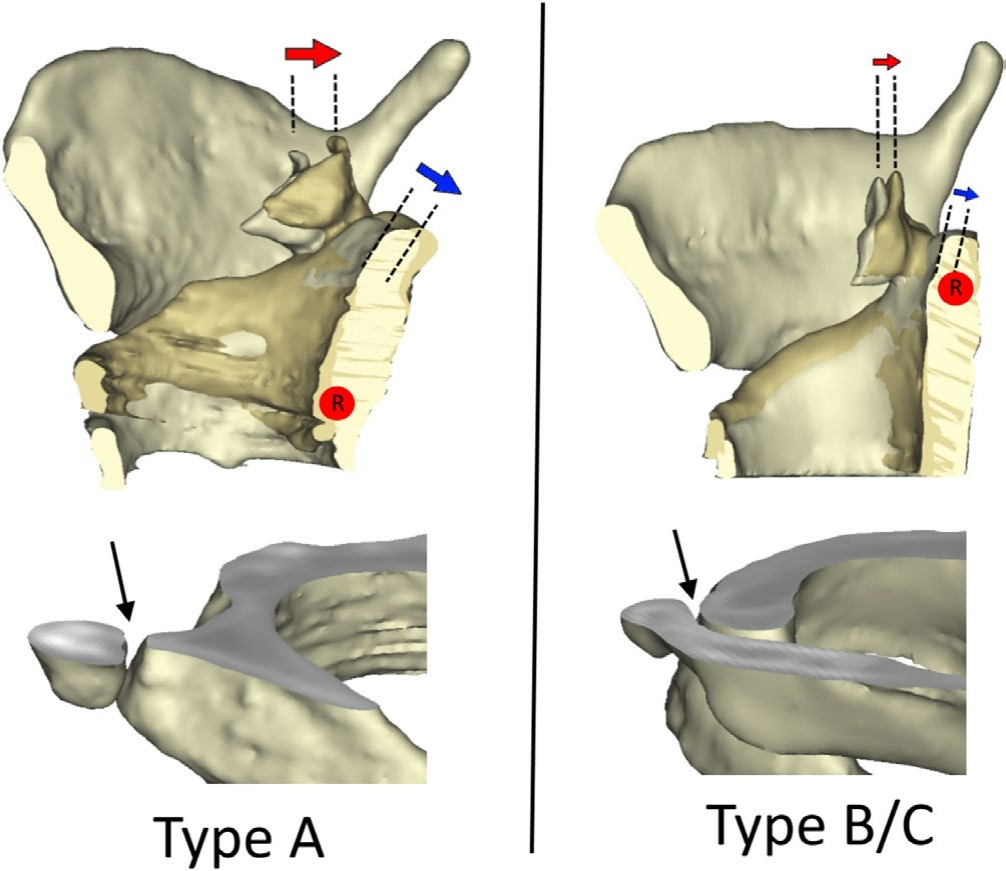
Above: View from the left of right hemilarynges of Type A and Type B/C with the preoperative and postoperative positions of the cricoid plate superimposed. Blue arrows indicate backward tilting of the cricoid plate; red arrows indicate posterior displacement of the arytenoid cartilages. Red dot: Rotation axis. Below: Right oblique frontal view of a three‐dimensional image of a larynx cut at the level of the cricothyroid joint. Type A shows the typical protuberance (arrow). In Type B/C, the cricoid part has a flat surface (arrow). [Color figure can be viewed in the online issue, which is available at www.laryngoscope.com]

In some cases, however, the voice cannot be raised sufficiently despite voice therapy, and this is when surgical options are indicated. Various techniques have been described in recent years to increase the vocal frequency, including reducing the vocal fold mass, shortening the vocal fold length, increasing the vocal fold tension, or combinations of these [4, 5, 6, 7, 8, 9, 10, 11, 12]. Cricothyroid approximation (CTA) aims to increase vocal fold tension by permanently fixing the cricoid and thyroid cartilage surgically. However, various studies have shown that although CTA initially achieves an increase in voice pitch, the voice frequency decreases again after several months [4, 5, 13, 14, 15]. Maue and Dickson [16] were the first to describe three different cricothyroid joint (CTJ) types: Type A describes a well‐defined articular surface on the cricoid with a tight joint capsule, with the joint protruding slightly from the cricoid; Type B shows a flat configuration on the cricoid with a cartilaginous articular surface and a loose joint capsule; and Type C shows no defined articular surface, with a loose joint capsule. About two‐thirds of CTJs are Type A, and one‐third are Type B or C [17] (Figure 1).

Storck was able to show that during the approximation of the cricoid to the thyroid cartilage, the rotation axis runs through both CTJs in a Type A CTJ joint. This results in greater vocal fold elongation due to a greater backward tilting of the cricoid and arytenoid cartilage than in a Type C CTJ, in which the virtual rotation axis runs through the upper part of the cricoid and thus results in much less vocal fold elongation [18]. Tschan and Storck showed that the Type A CTJ has a direct influence on vocal fold elongation and thus on pitch raising in CTA (Figure 1) [19]. Patients with a stable Type A CTJ showed a significantly higher speaking voice frequency postoperatively than those with an unstable Type B/C CTJ [19]. Therefore, a CTA should be performed only in trans women with a Type A CTJ [19].

In the studies published to date, all patients with a Type A CTJ or Type B/C CTJ were included. We postulate that this may explain the sometimes inconsistent follow‐up voice frequencies, as Type B/C CTJs are probably those in whom the voice deepens again and thus influences the overall results.

Current data suggest that a satisfactory outcome can be expected only in patients with a Type A CTJ. Tschan et al. have, therefore, recommended that CTA, which is generally a very safe surgical technique without serious complications, should be performed only in patients with a Type A CTJ [19].

Therefore, the aim of our study was to perform a follow‐up after CTA surgery in trans women with a stable Type A CTJ to determine whether the MSL remains significantly elevated up to 5 years after surgery compared with the preoperative state. Furthermore, we assessed voice satisfaction by means of a questionnaire (Trans Woman Voice Questionnaire [TWVQ]) [20, 21].

## 2 Materials and Methods

### 2.1 Study Population

A total of 44 trans women underwent CTA at the University Hospital of Basel, between 2009 and 2022. All patients underwent laryngostroboscopic examination to rule out organic findings. Additionally, all patients underwent high‐resolution computed tomography (HRCT) of the larynx prior to surgery to determine the CTJ type [22]. Of these, 35 showed a Type A CTJ and were included in the study. The mean age of the participants was 41 years (17–75 years). Every patient had voice therapy first. On average, they attended 14 [4, 5, 6, 7, 8, 9, 10, 11, 12, 13, 14, 15, 16, 17, 18, 19, 20, 21, 22, 23, 24] voice therapy sessions prior to surgery, yet the results remained unsatisfactory: their voices were inadequately elevated and the patients were dissatisfied with them. Among the reasons given were that the voice pitch did not stay high enough and that it was too strenuous to speak at a high pitch; that patients always had to remember to keep their voice pitch high; that in the morning the voice pitch was high enough, but toward the afternoon or evening the voice pitch declined; and that with vocal reflexes, the voice pitch was low and could not be influenced. To further improve voice pitch elevation, all patients desired surgical intervention. None of the patients was an amateur or professional singer.

### 2.2 Methods

All CTAs were done under general anesthesia by the same surgeon (C.S.). The procedures were performed using two titanium miniplates as proposed by Neumann et al. [4] to prevent loosening of the sutures after surgery.

To determine the mean speaking level (MSL) vocal range and volume, a speaking voice range profile was recorded from all patients before and after voice therapy, 4 weeks postoperatively (on average 35 days postoperatively), 6 months postoperatively (on average 193 days postoperatively), and then annually until 5 years after surgery. The speaking voice range profile was recorded using the voice analysis software Lingwaves and by having the patients read the standardized text “The North Wind and the Sun” from Aesop's Fables. The recordings were performed according to the recommendations of the Union of European Phoniatrics under normal room acoustics [23].

During the session when the speaking voice range profiles were recorded, the authorized German version [21, 24] of the TWVQ [20, 25] was completed to measure the voice‐related impacts on the patients' daily lives.

The statistical analysis was performed by Flurin Honegger, using the nonparametric statistical test developed by Milton Friedman and the post hoc comparison with Conover's all‐pairs comparison tests of Friedman‐type ranked data (Bonferroni). The study was approved by the Medical Ethics Committee of the University of Basel, Switzerland.

## 3 Results

The observations of all 35 patients included in the study were conducted until 6 months after CTA. Unfortunately, during the following period, some of the patients were lost to follow‐up, mainly due to personal circumstances. Data were available from 23 patients after 1 year, 15 patients after 2 years, 13 patients after 3 years, 8 patients after 4 years, and 4 patients after 5 years. Out of 35 patients, 12 stated that they smoked occasionally (1–2 times a week). The laryngoscopic findings were normal in all patients; only one patient showed edema of both vocal folds due to a history of heavy smoking (50 py). Medication that could impair vocal quality was denied by all patients.

### 3.1 MSL

**FIGURE 2:**
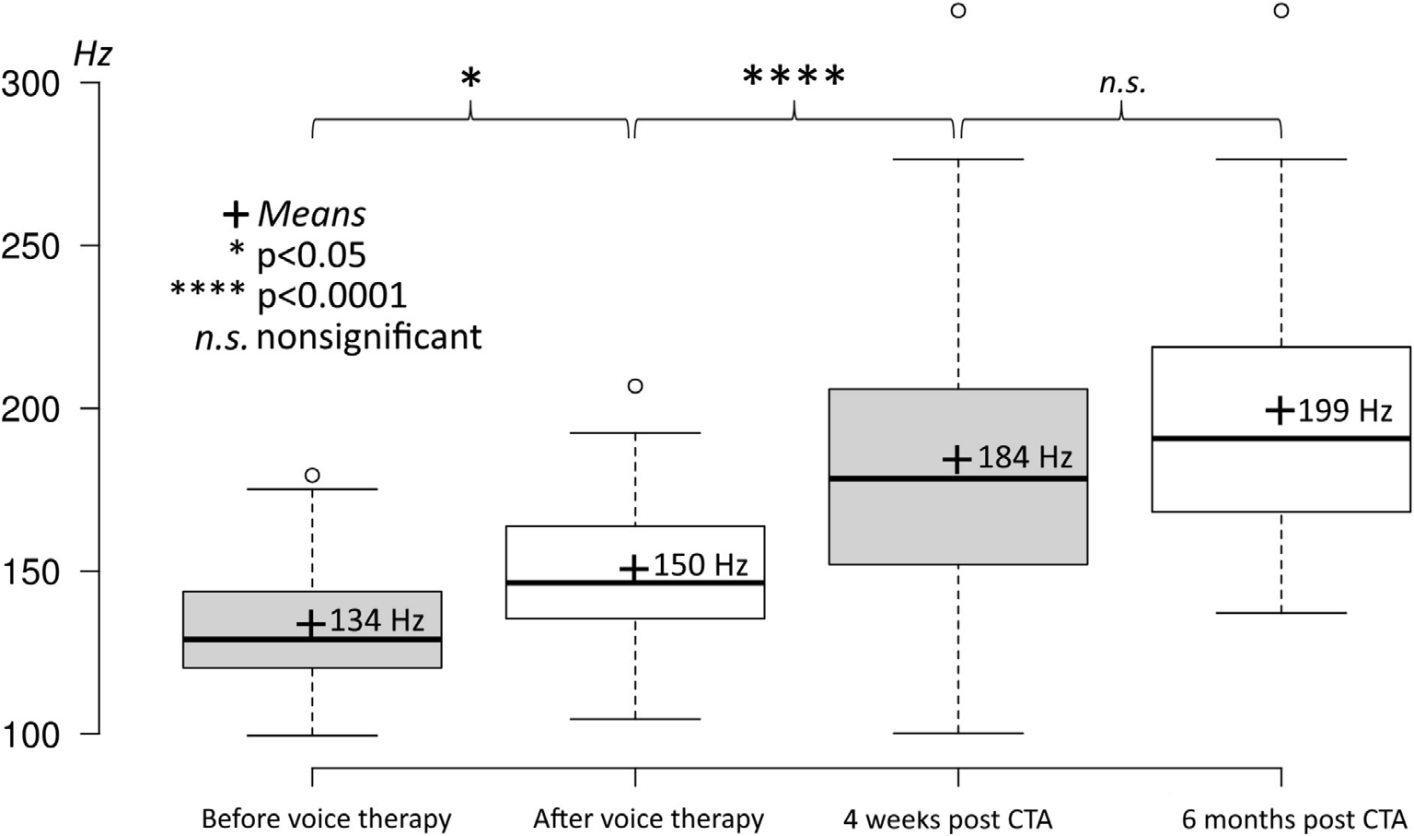
Mean speaking level (MSL) over the period from before voice therapy to 6 months post operation. There was a significant elevation of MSL after CTA.

**FIGURE 3:**
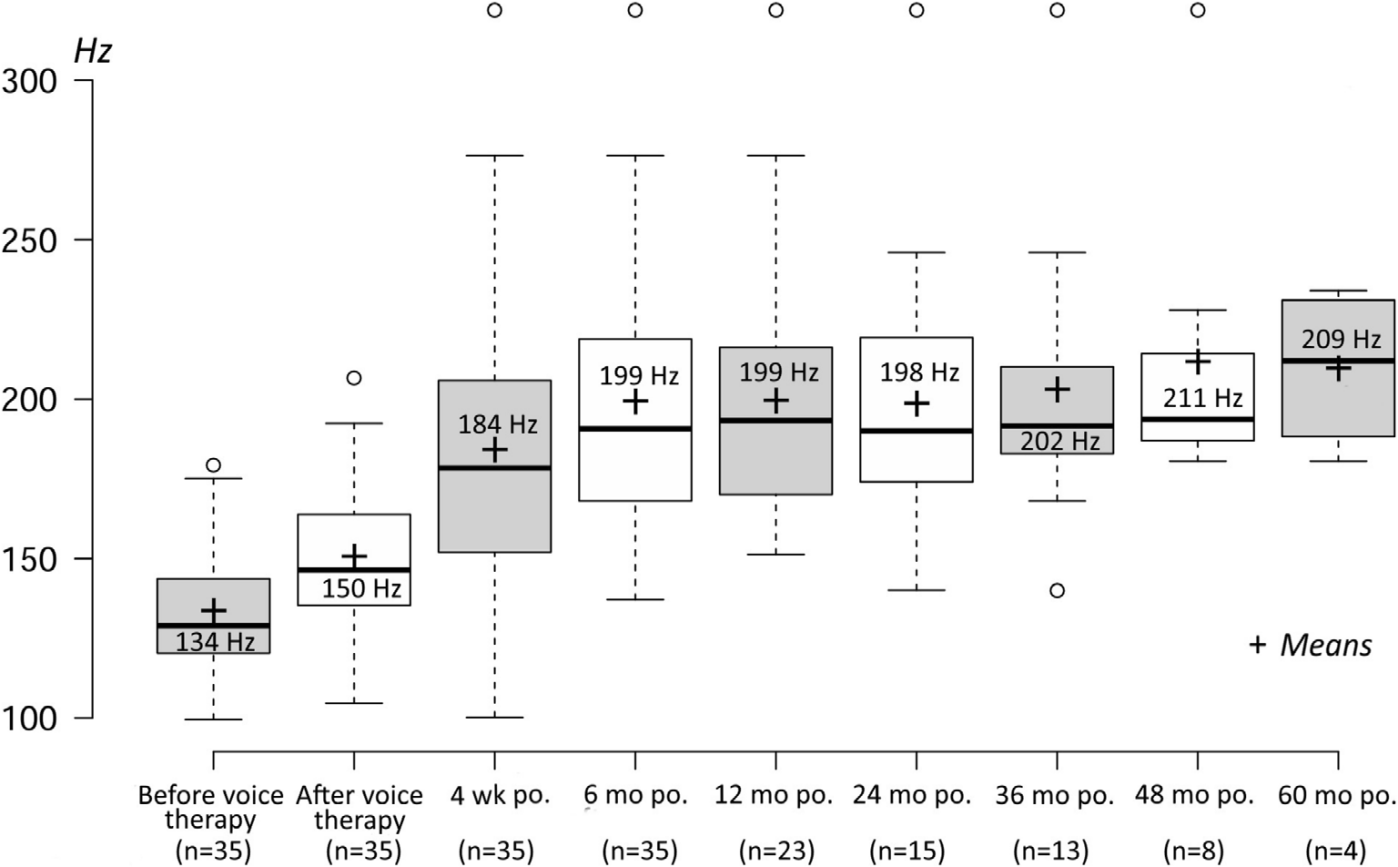
Mean speaking level (MSL) across the whole 5‐year follow‐up. The postoperative MSL remained stable and did not decline.

Prior to voice therapy, the MSL was at 134 Hz (range: 100–179 Hz); after voice therapy, the MSL increased significantly to 150 Hz (range: 105–207 Hz). Four weeks after CTA, the mean MSL had increased to 184 Hz (*n* = 35; range: 100–322 Hz). Six months after the procedure, MSL had increased once again, but not significantly, to 199 Hz (*n* = 35; range: 137–322 Hz) (Figure 2). One year after CTA, the mean MSL remained significantly elevated at 199 Hz (*n* = 23; range: 151–322 Hz). Two years after CTA, the mean MSL remained significantly elevated at 198 Hz (*n* = 15; range: 140–322 Hz). Three years after CTA, a trend was observed with a mean MSL of 203 Hz (*n* = 13; range: 140–322 Hz); 4 years after CTA, the mean MSL was 211 Hz (*n* = 8; range: 180–322 Hz); and 5 years after CTA, the mean MSL was 209 Hz (*n* = 4; range: 180–234 Hz). As the patient groups were too small 3 years post operation, we did not calculate any significance, but we were able to observe a trend that MSL remained stably high (Figure 3). One patient, who was a very heavy smoker with small Reinke edema, was seen laryngoscopically; no pitch elevation was achieved with the CTA. This is shown in Figures 2 and 3.

### 3.2 Speaking Voice Volume

The speaking volume did not change after the surgery, with a mean voice volume of 70 dB (range: 58–82 dB) prior to surgery, 70 dB (range: 58–86 dB) measured postoperatively (n.s.), and 69 dB (range: 61–82 dB) 6 months after the procedure. The minimum speech volume changed from 59 dB (range: 43–72 dB) measured preoperatively to 57 dB (range: 42–74 dB) measured postoperatively and 56 dB (range: 42–69 dB) 6 months after CTA. The maximum volume also remained stable from 78 dB (range: 65–91 dB) measured preoperatively to 77 dB (range: 66–92 dB) measured postoperatively and 78 dB (range: 64–97 dB) 6 months after CTA.

### 3.3 Vocal Range of the Speaking Voice

**FIGURE 4:**
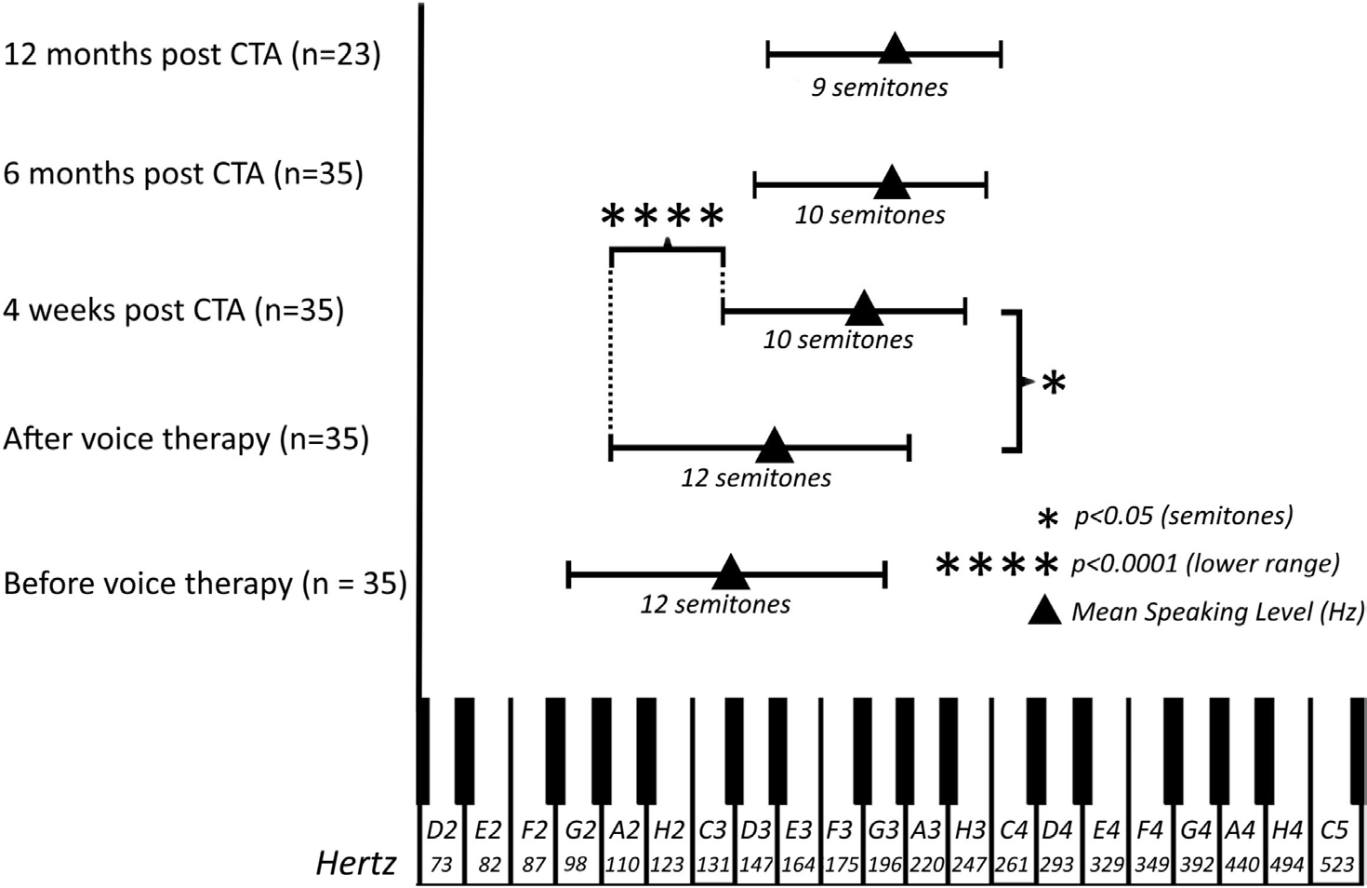
Vocal range of the speaking voice from before voice therapy to 6 months post operation. There was a significant reduction in semitones and especially of the lower limit after CTA.

Before voice therapy, the mean vocal range was 12 semitones (range: 6–21 semitones) with a mean value of the lowest tone of 95 Hz (range: 54–143 Hz) and a mean value of the highest of 191 Hz (range: 120–267 Hz). After voice therapy, the vocal range did not change. It remained at a mean of 12 semitones (range: 5–23 semitones); the mean lowest tone was 104 Hz (60–159 Hz), and the mean highest tone was 210 Hz (range: 134–297 Hz). Four weeks post operation, the vocal range was significantly lower (*p* < 0.05) by two semitones to an average of 10 semitones (range: 4–21 semitones). The lower limit was 141 Hz (range: 88–277 Hz), and the upper limit was 248 Hz (range: 143–466 Hz). The reduction in vocal range was due to the highly significantly increased (*p* < 0.0001) lower limit of the vocal range after CTA. Six months post operation, the mean vocal range remained at 10 semitones (range: 6–21 semitones). The mean lower limit of the vocal range was 149 Hz (99–277 Hz), while the mean upper limit was 266 Hz (189–466 Hz). Even 12 months post operation, the values had not changed: the mean vocal range was 9 semitones (range: 6–21 semitones), the mean lower limit was 154 Hz (range: 120–277 Hz), and the mean upper limit was 263 Hz (range: 196–466 Hz) (Figure 4). After the CTA, voice therapy was recommended to all the patients. Of 35 patients, only 15 made use of this. These were mainly the patients with the lower speaking voices.

### 3.4 Voice Range of the Singing Voice

The vocal range of the singing voice was also measured. Before voice therapy, the lower limit was 82 Hz (range 65–110 Hz), and the upper limit was 289 Hz (range 260–340 Hz). The vocal range was 207 Hz (22 semitones), while the average voice dynamic ranged from 57 (range 54–60 dB) to 85 (range 78–91 dB) (mean 27 dB) (range 52–64 dB) to 87 dB (79–98 dB) (mean 29 dB). The voice therapy did not significantly change the vocal range and voice dynamics; only the vocal field was shifted slightly to the right into the higher vocal range.

Six months postoperatively, the lower limit was 123 Hz (range 98–140 Hz), and the upper limit was 349 Hz (range 260–389 Hz). The vocal range was 226 Hz (18 semitones), while the average voice dynamic ranged from 60 dB (range 50–66 dB) to 86 dB (76–95 dB) (mean 26 dB).

**FIGURE 5:**
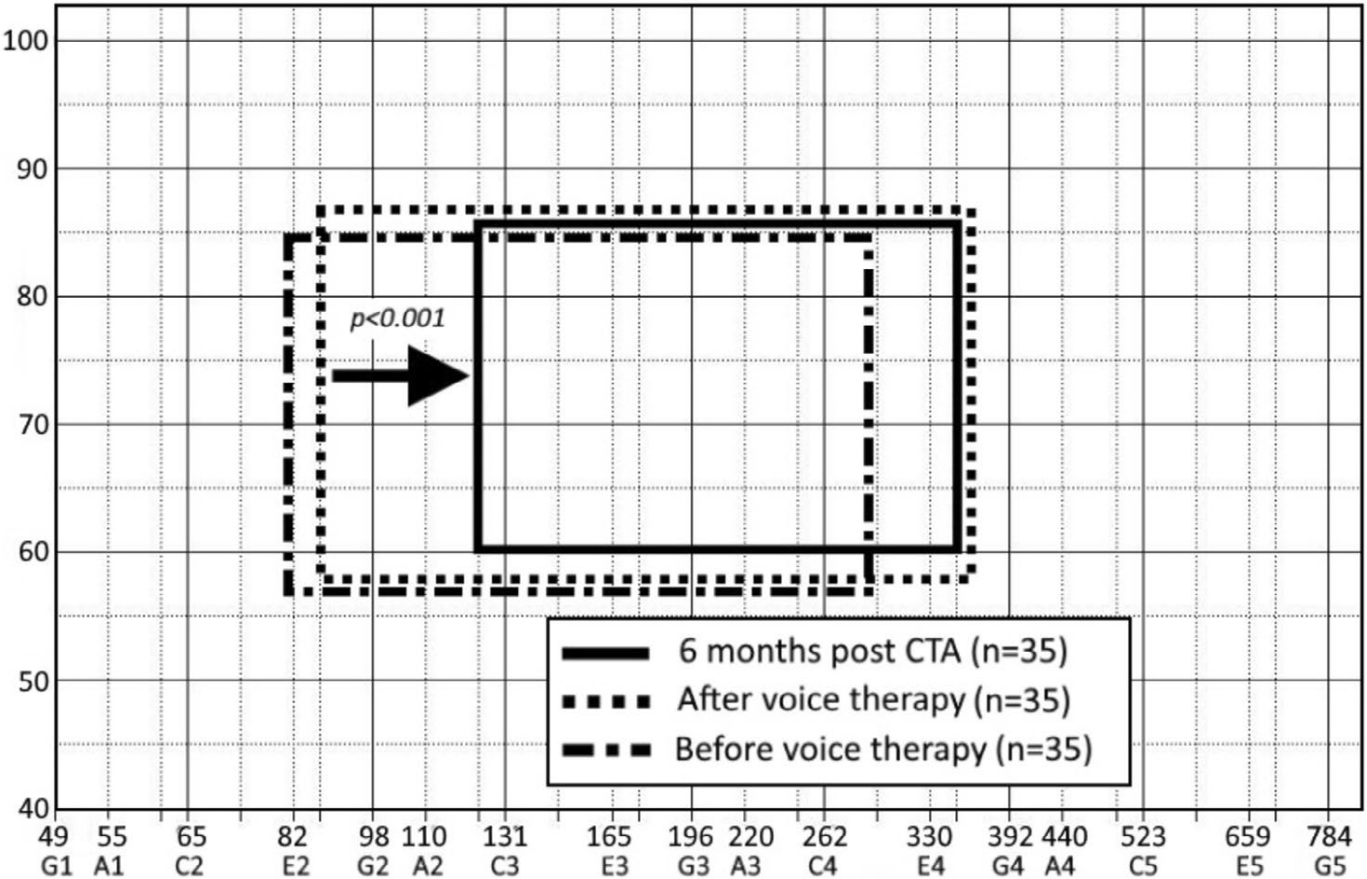
Schematic depiction of the singing voice range before and after voice therapy and after cricothyroid approximation. There was a significant shift of the lower limit with a reduction of the singing range.

The CTA significantly (*p* < 0.001) reduced the vocal range by shifting the lower limit of the vocal range. The voice dynamics did not decrease significantly (Figure 5).

### 3.5 TWVQ

**FIGURE 6:**
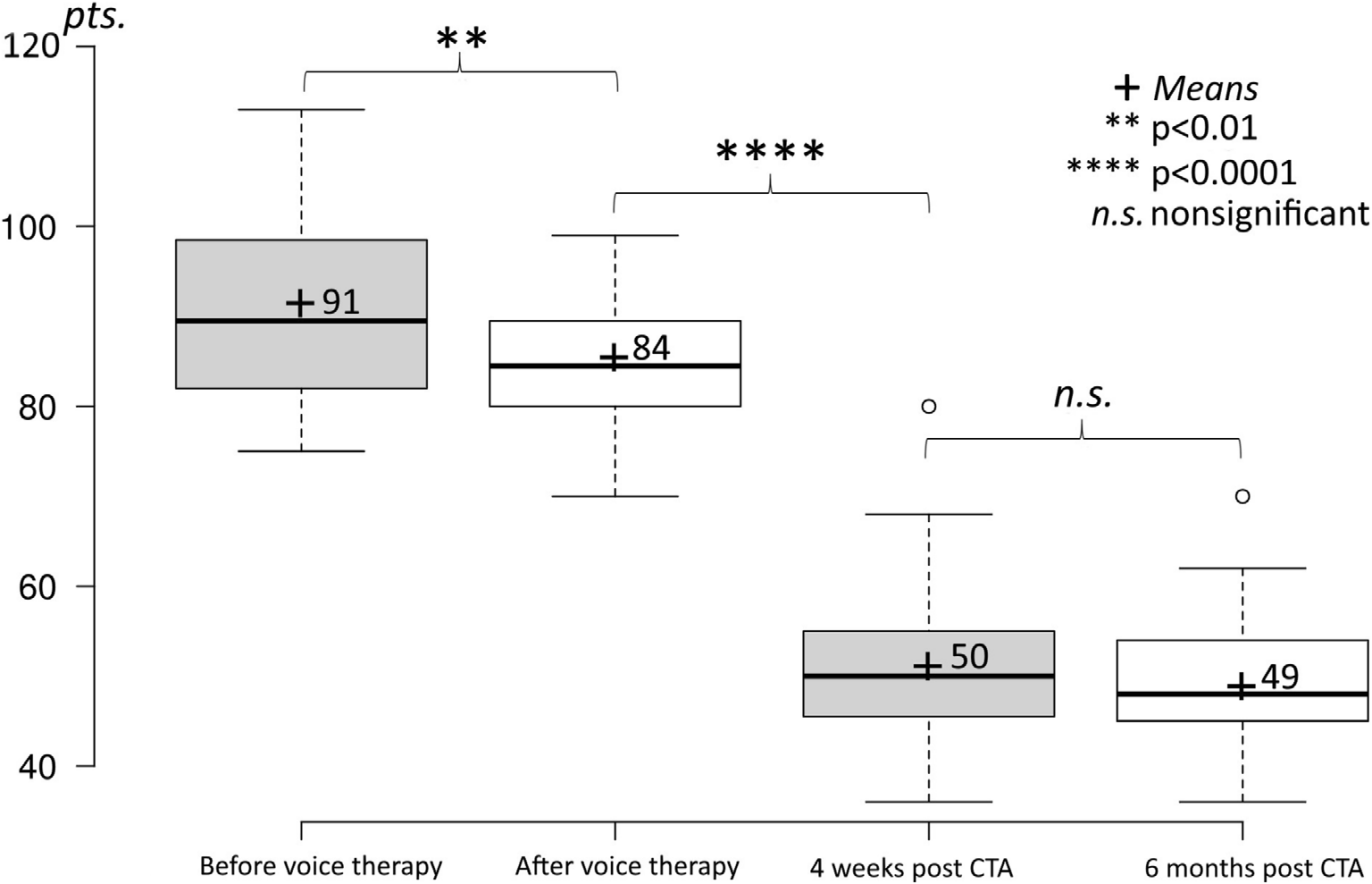
TWVQ values from before voice therapy to 6 months post operation. There was a significant reduction with CTA.

The mean TWVQ score prior to voice therapy was 91 points (range: 75–113), and it was significantly (*p* < 0.01) lower after voice therapy, at 84 points (range: 70–99). After CTA, the TWVQ was very significantly lower, at 50 points (range: 36–80) (*p* < 0.0001). This remained stable until 6 months post operation, at 49 points (range: 36–70) (Figure 6). During the follow‐up period, the value decreased annually until it reached 48 points (range: 36–62) 3 years after CTA. In the subsequent annual measurements at 4 and 5 years, the TWVQ value did not change significantly in the smaller patient groups (not depicted in Figure 6).

## 4 Discussion

Trans women may not always be perceived as female because of their voice characteristics. When nonsurgical methods such as voice therapy do not yield the desired results, surgical intervention becomes an option. A common surgical approach is CTA, first described by Isshiki et al. [6], which involves increasing the tension of the vocal folds to raise the vocal pitch. Many studies have examined the outcomes of CTA [5, 15, 26, 27, 28], but none of these studies focused on patients with Type A CTJ. As shown in a previous study [19], the success of CTA surgery depends on the patient's CTJ type. This is because the tilting of the cricoid, which leads to the lengthening and tensioning of the vocal fold and consequently to the elevation of the vocal pitch, is sufficient only in Type A joints [18]. Therefore, in our study, we included only patients who were revealed to have Type A joints in the preoperative HRCT.

Our study yielded the following results: (1) MSL can be improved by means of voice therapy voice training. The majority of patients reach the gender‐neutral vocal range. (2) CTA of the Type A joint leads to a higher MSL, in the feminine vocal range, by approximately 50 Hz (five semitones). As a result of the operation, the MSL remains stable and does not drop over a follow‐up of 5 years. (3) The voice volume remains the same postoperatively, without becoming softer. (4) CTA reduces the vocal range in the lower frequency range. The upper frequency range is extended by voice therapy. (5) Postoperatively, patient satisfaction is significantly better and remains so.

Or results show that voice therapy can increase MSL into the gender‐neutral voice range, although this change is not significant. On the other hand, there is a significant reduction of TWVQ score. This means that voice therapy has a positive influence on the self‐perception of the speaking voice [2, 5, 29], but probably not enough for the speaker to really be perceived as a woman [30].

The CTA in patients with Type A CTJ shows a significant increase in the MSL. In particular, the MSL has remained stably high over a period of 5 years and has not decreased, although the observed group has become smaller. This shows that CTA is a good way to raise the MSL in trans women with a Type A CTJ without it dropping again within months [5, 13]. Our data confirm the observations of Tschan et al. in which we compared the MSL after CTA for the different joint types (Type A vs. Type B/C). This showed that patients with a Type A joint had a significantly greater increase in MSL than patients with a Type B/C joint. Thus, the selection of patients on the basis of an HRCT of the larynx for the evaluation of a Type A CTJ is decisive as to whether CTA will be successful [19]. The increase in pitch speaking voice within the first 6 months is certainly due to both the scarring and shortening of the cricothyroid muscle that increase the tension of the vocal folds and to the patients' voice therapy. Therefore, we also recommend voice therapy after surgery [31]. This allows the patient to become familiar with their new voice pitch, refine it, and identify more with it.

It is important to note that previous studies on CTA included all patients, regardless of whether they had a Type A, Type B, or Type C CTJ, because it was not yet known that the joint type can significantly influence the MSL. This also explains why most studies mention that the MSL deepens again after a short observation period [4, 5]. Due to the instability of the Type C CTJ capsule, long‐lasting voice elevation is not guaranteed. This also explains the dissatisfaction after CTA of around one‐third of the patients.

As is generally assumed, in our study the speaking voice volume did not become softer. This is probably due to the fact that the mass of the vocal folds remains the same, and the tension probably has no influence on the speaking voice volume. In contrast to CTA, vocal fold mass is reduced in glottoplasty, laser‐assisted voice adjustment, and feminization laryngoplasty, and therefore a reduced speaking voice volume can be expected [9, 32, 33, 34]. In addition, our data show that the vocal range of the singing voice is significantly reduced by six semitones after CTA, especially in the lower limit of the singing voice. The majority of patients stated that they very much appreciated the fact that the low frequency ranges were no longer present. In particular, the otherwise low voice could no longer be reproduced during reflexes such as laughing, crying, whooping, or coughing. Our data clearly show that the lower part of the vocal range was reduced significantly (Figure 4). This is clearly related to the fact that these frequencies could no longer be reached due to the increased tension of the vocal folds. It is wrong to assume that CTA also increases the upper frequency ranges. The high frequency range is probably a question of vocal training.

However, the vocal range of the speaking voice was significantly reduced as a result. Interestingly, however, every patient stated that they did not feel that their voice sounded monotonous. This was probably also the result of pre‐ and postoperative voice therapy, by which patients learn to train a melodious speaking voice.

In principle, however, we do not recommend CTA for patients who enjoy singing as a hobby or professionally, as the cricothyroid muscle is responsible for singing in the chest voice from the mean singing voice frequency up to the first octave [35], and this operation eliminates the function of the cricothyroid muscle. Overall, CTA significantly improved the TWVQ score, and this had a clearly positive effect on the patients' passing as female.

In three patients, MSL was still low even after CTA. One patient who was a heavy smoker had altered vocal folds on both sides. In two other patients, vocal fold length and mass probably played a role. It is plausible that not only must a stable CTJ be present for optimal voice results after CTA, but the vocal fold length and mass must also not be too large, as otherwise the CTA will not be sufficient to generate enough tension on the vocal folds. In these cases, an additional voice‐feminizing operation would then be suggested. In our two such cases, the patients did not appear for further examinations.

In the case of heavy smokers, it is to be expected that despite an increase in the tension of the vocal folds, the pitch will not increase. This can probably be explained by the fact that, on the one hand, the vocal fold mucosa is altered by smoking and on the other hand, tissue changes cause a mass enlargement of the vocal folds and therefore no increase in the voice pitch is possible. We therefore recommend that heavy smokers do not undergo CTA.

Our study has a few limitations. We lost some patients to follow‐up for various reasons. Some changed their place of residence, and two others could no longer be contacted. These patients may not have been completely satisfied with the postoperative MSL and therefore no longer came for check‐ups. If so, as a result, we included only those patients who were really satisfied. However, this is purely hypothetical. Another limitation is that we did not measure prosody, which can be limited by CTA.

## 5 Conclusion

To the best of our knowledge, this is the first study to show that CTA can be successful in selected patients. We can state that a significant increase in MSL can be achieved with CTA in patients with a Type A CTJ and that this voice pitch is maintained. The satisfaction of the patients is also good. Therefore, if a CTA is to be considered, an HRCT of the larynx should be performed beforehand with special attention to the CTJ. CTA should generally be avoided in patients who like to sing or who are heavy smokers.

## Conflicts of Interest

The authors declare no conflicts of interest.

## References

[1] W. Bockting , E. Coleman , M. B. Deutsch , et al., “Adult Development and Quality of Life of Transgender and Gender Nonconforming People,” Current Opinion in Endocrinology, Diabetes, and Obesity 23 (2016): 188–197. 26835800 10.1097/MED.0000000000000232 PMC4809047

[2] A. B. Hancock , J. Krissinger , and K. Owen , “Voice Perceptions and Quality of Life of Transgender People,” Journal of Voice 25 (2011): 553–558. 21051199 10.1016/j.jvoice.2010.07.013

[3] B. Nuyen , C. Kandathil , D. McDonald , J. Thomas , and S. P. Most , “The Impact of Living With Transfeminine Vocal Gender Dysphoria: Health Utility Outcomes Assessment,” International Journal of Transgender Health 24 (2023): 99–107. 36713148 10.1080/26895269.2021.1919277 PMC9879186

[4] K. Neumann , C. Welzel , and A. Berghaus , “Operative Voice Pitch Raising in Male‐to‐Female Transsexuals. A Survey of Our Technique and Results,” HNO 51 (2003): 30–37. 12557095 10.1007/s00106-002-0654-4

[5] C. Y. Yang , A. D. Palmer , K. D. Murray , T. R. Meltzer , and J. I. Cohen , “Cricothyroid Approximation to Elevate Vocal Pitch in Male‐to‐Female Transsexuals: Results of Surgery,” Annals of Otology, Rhinology, and Laryngology 111 (2002): 477–485. 12090702 10.1177/000348940211100602

[6] N. Isshiki , H. Morita , H. Okamura , and M. Hiramoto , “Thyroplasty as a New Phonosurgical Technique,” Acta Oto‐Laryngologica 78 (1974): 451–457. 4451096 10.3109/00016487409126379

[7] N. Isshiki , H. Morita , H. Okamura , and M. Hirarnoto , Experimental and Clinical Study of Thyroplasty as a New Type of Phonosurgery (S Karger, 1976), 213–216.

[8] N. Isshiki , T. Taira , and M. Tanabe , “Surgical Alteration of the Vocal Pitch,” Journal of Otolaryngology 12 (1983): 335–340. 6644864

[9] J. Wendler , “Vocal Pitch Elevation After Transsexualism Male to Female,” in *Proceedings of the Union of the European Phoniatricians* (1990).

[10] M. Gross , “Pitch‐Raising Surgery in Male‐to‐Female Transsexuals,” Journal of Voice 13 (1999): 246–250. 10442755 10.1016/s0892-1997(99)80028-9

[11] F. E. LeJeune , C. E. Guice , and P. M. Samuels , “Early Experiences With Vocal Ligament Tightening,” Annals of Otology, Rhinology, and Laryngology 92 (1983): 475–477. 6625446 10.1177/000348948309200513

[12] H. M. Tucker , “Anterior Commissure Laryngoplasty for Adjustment of Vocal Fold Tension,” Annals of Otology, Rhinology, and Laryngology 94 (1985): 547–549. 4073730 10.1177/000348948509400604

[13] J. A. Koufman and G. Isaacson , “Laryngoplastic Phonosurgery,” Otolaryngologic Clinics of North America 24 (1991): 1151–1177. 1754218

[14] M. Brown , A. Perry , A. D. Cheesman , and T. Pring , “Pitch Change in Male‐to‐Female Transsexuals: Has Phonosurgery a Role to Play?,” International Journal of Language & Communication Disorders 35 (2000): 129–136. 10824229 10.1080/136828200247296

[15] E. Mora , I. Cobeta , A. Becerra , and M. J. Lucio , “Comparison of Cricothyroid Approximation and Glottoplasty for Surgical Voice Feminization in Male‐to‐Female Transsexuals,” Laryngoscope 128 (2018): 2101–2109. 29573435 10.1002/lary.27172

[16] W. M. Maue and D. R. Dickson , “Cartilages and Ligaments of the Adult Human Larynx,” Archives of Otolaryngology 94 (1971): 432–439. 5114952 10.1001/archotol.1971.00770070678008

[17] G. P. Hammer , G. Windisch , P. M. Prodinger , F. Anderhuber , and G. Friedrich , “The Cricothyroid Joint—Functional Aspects With Regard to Different Types of Its Structure,” Journal of Voice 24 (2010): 140–145. 19185450 10.1016/j.jvoice.2008.07.001

[18] C. Storck , R. Gehrer , C. Fischer , et al., “The Role of the Cricothyroid Joint Anatomy in Cricothyroid Approximation Surgery,” Journal of Voice 25 (2011): 632–637. 20971613 10.1016/j.jvoice.2010.06.001

[19] S. Tschan , F. Honegger , and C. Storck , “Cricothyroid Joint Anatomy as a Predicting Factor for Success of Cricoid‐Thyroid Approximation in Transwomen,” Laryngoscope 126 (2016): 1380–1384. 26227170 10.1002/lary.25518

[20] G. Dacakis , S. Davies , J. M. Oates , J. M. Douglas , and J. R. Johnston , “Development and Preliminary Evaluation of the Transsexual Voice Questionnaire for Male‐to‐Female Transsexuals,” Journal of Voice 27 (2013): 312–320. 23415146 10.1016/j.jvoice.2012.11.005

[21] G. Dacakis and J. Oates , “Trans Woman Voice Questionnaire (Formerly TVQMtF),” 2012, https://www.latrobe.edu.au/__data/assets/pdf_file/0010/1393363/TGV‐Resources.pdf.

[22] J. Koch , F. Unteregger , F. Honegger , S. Potthast , and C. Storck , “The Cricothyroid Joint: A Practical Guide for Distinguishing Between Different Joint Types,” Journal of Voice 34 (2020): 33–37. 30245213 10.1016/j.jvoice.2018.08.019

[23] H. K. Schutte and W. Seidner , “Recommendation by the Union of European Phoniatricians (UEP): Standardizing Voice Area Measurement/Phonetography,” Folia Phoniatrica 35 (1983): 286–288. 6654278 10.1159/000265703

[24] S. Salm , K. Hower , S. Neumann , and L. Ansmann , “Validation of the German Version of the Transsexual Voice Questionnaire for Male‐to‐Female Transsexuals,” Journal of Voice 34 (2020): 68–77. 30172668 10.1016/j.jvoice.2018.06.010

[25] G. Dacakis , J. M. Oates , and J. M. Douglas , “Further Evidence of the Construct Validity of the Transsexual Voice Questionnaire (TVQ(MtF)) Using Principal Components Analysis,” Journal of Voice 31 (2017): 142–148. 27515560 10.1016/j.jvoice.2016.07.001

[26] S. Van Damme , M. Cosyns , S. Deman , Z. Van den Eede , and J. Van Borsel , “The Effectiveness of Pitch‐Raising Surgery in Male‐to‐Female Transsexuals: A Systematic Review,” Journal of Voice 31 (2017): 244. 10.1016/j.jvoice.2016.04.002 27474996

[27] J. Kanagalingam , C. Georgalas , G. R. Wood , S. Ahluwalia , G. Sandhu , and A. D. Cheesman , “Cricothyroid Approximation and Subluxation in 21 Male‐to‐Female Transsexuals,” Laryngoscope 115 (2005): 611–618. 15805869 10.1097/01.mlg.0000161357.12826.33

[28] K. Neumann and C. Welzel , “The Importance of the Voice in Male‐to‐Female Transsexualism,” Journal of Voice 18 (2004): 153–167. 15070236 10.1016/S0892-1997(03)00084-5

[29] G. Dacakis , J. Oates , and J. Douglas , “Associations Between the Transsexual Voice Questionnaire (TVQ^MtF^) and Self‐Report of Voice Femininity and Acoustic Voice Measures,” International Journal of Language & Communication Disorders 52 (2017): 831–838. 28425220 10.1111/1460-6984.12319

[30] J. Van Borsel , E. Van Eynde , G. De Cuypere , and K. Bonte , “Feminine After Cricothyroid Approximation?,” Journal of Voice 22 (2008): 379–384. 17280818 10.1016/j.jvoice.2006.11.001

[31] Y. Leung , J. Oates , and S. P. Chan , “Voice, Articulation, and Prosody Contribute to Listener Perceptions of Speaker Gender: A Systematic Review and Meta‐Analysis,” Journal of Speech, Language, and Hearing Research 61 (2018): 266–297. 10.1044/2017_JSLHR-S-17-0067 29392290

[32] L. A. Orloff , A. P. Mann , J. F. Damrose , and S. N. Goldman , “Laser‐Assisted Voice Adjustment (LAVA) in Transsexuals,” Laryngoscope 116 (2006): 655–660. 16585875 10.1097/01.mlg.0000205198.65797.59

[33] B. A. Nuyen , Z. J. Qian , R. D. Campbell , E. Erickson‐DiRenzo , J. Thomas , and C. K. Sung , “Feminization Laryngoplasty: 17‐Year Review on Long‐Term Outcomes, Safety, and Technique,” Otolaryngology and Head and Neck Surgery 167 (2022): 112–117. 10.1177/01945998211036870 34399638

[34] J. P. Thomas and C. Macmillan , “Feminization Laryngoplasty: Assessment of Surgical Pitch Elevation,” European Archives of Oto‐Rhino‐Laryngology 270 (2013): 2695–2700. 23632870 10.1007/s00405-013-2511-3

[35] F. Unteregger , F. Honegger , S. Potthast , S. Zwicky , J. Schiwowa , and C. Storck , “3D Analysis of the Movements of the Laryngeal Cartilages During Singing,” Laryngoscope 127 (2017): 1639–1643. 27882556 10.1002/lary.26430

